# Atmospheric wave energy of the 2020 August 4 explosion in Beirut, Lebanon, from ionospheric disturbances

**DOI:** 10.1038/s41598-021-82355-5

**Published:** 2021-02-02

**Authors:** Bhaskar Kundu, Batakrushna Senapati, Ai Matsushita, Kosuke Heki

**Affiliations:** 1 Department of Earth and Atmospheric Science, NIT Rourkela, Rourkela, 769008 India; 2 Department of Earth and Planetary Science, Hokkaido University, Sapporo, Hokkaido 060-0810 Japan

**Keywords:** Geophysics, Aurora, Magnetospheric physics

## Abstract

Atmospheric waves excited by strong surface explosions, both natural and anthropogenic, often disturb upper atmosphere. In this letter, we report an N-shaped pulse with period ~ 1.3 min propagating southward at ~ 0.8 km/s, observed as changes in ionospheric total electron content using continuous GNSS stations in Israel and Palestine, ~ 10 min after the August 4, 2020 chemical explosion in Beirut, Lebanon. The peak-to-peak amplitude of the disturbance reached ~ 2% of the background electrons, comparable to recently recorded volcanic explosions in the Japanese Islands. We also succeeded in reproducing the observed disturbances assuming acoustic waves propagating upward and their interaction with geomagnetic fields.

## Introduction

**Figure 1:**
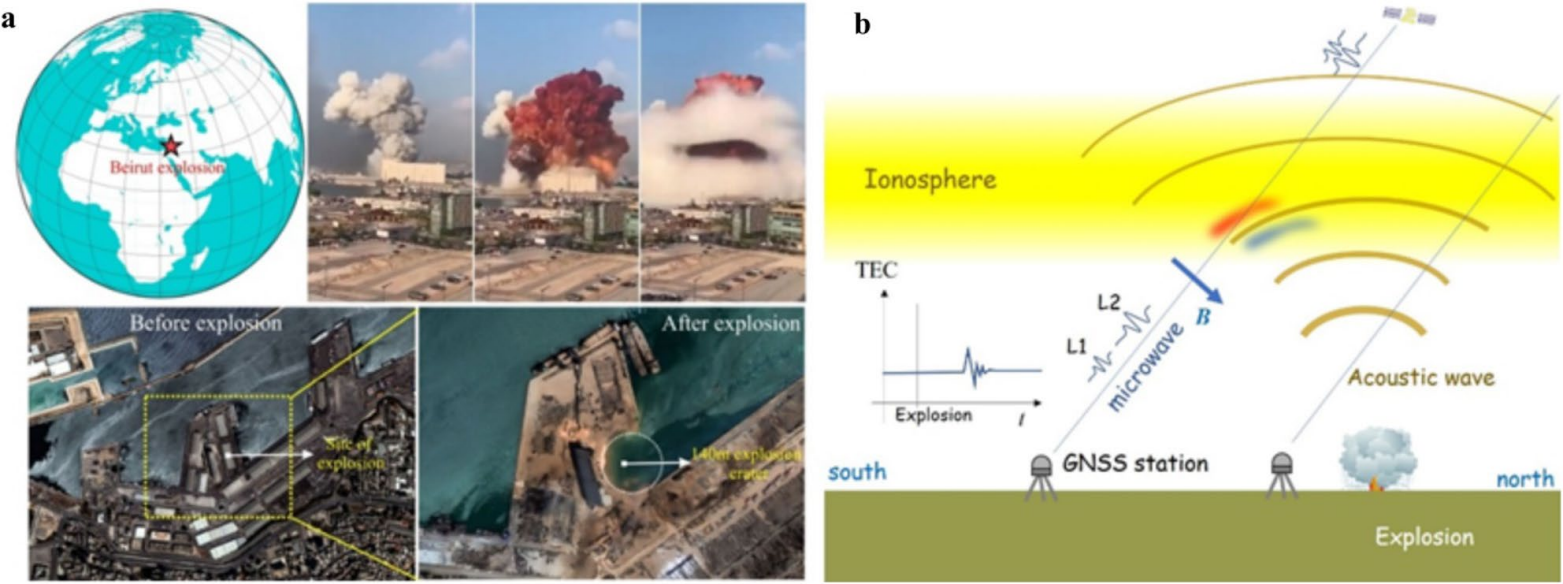
The 2020 Beirut explosion and ionospheric disturbance. (**a**) Location of August 4, 2020 Beirut explosion point marked by a red star in the globe. Sequence of the ash cloud captured during the explosion (snaps archived at https://www.thenewsminute.com/article/shocking-videos-show-powerful-explosion-rocks-lebanon-capital-beirut-130041). SkySat imagery before and after the explosion shows the impact of explosion in Beirut (imagery captured on May 31, 2020 and on August 5, 2020) (archived at the largest earth-observation-satellite-network of global dataset platform at https://www.theguardian.com/world/2020/aug/06/beirut-explosion-before-and-after-satellite-images). (**b**) Illustration of ionospheric disturbance caused by an explosion can be detected by differential ionospheric delays of microwave signals of two carrier frequencies from GNSS satellites. The disturbances in ionosphere are observed 10–12 min after the explosion, the time necessary for the acoustic wave to reach the ionospheric F region. The wave makes electron density anomalies (a pair of positive and negative anomalies shown with red and blue) on the southern side of the explosion. Figure (**a**,**b**) were generated using Corel Draw (version 18) graphical application (URL: https://www.coreldraw.com/en ).

Beirut, known as Paris of Middle East, is an ancient capital of Lebanon in the eastern Mediterranean coast, scarred by civil war, and home of ~ 2 million residents. The socio-economic backbone of the city became paralysed by a deadly explosion on August 4, 2020 at a warehouse in the port, where > 2750 tonnes of ammonium nitrate (equivalent to ~ 1.1 kt of TNT) was stored without proper safety measures. The largest explosion occurred at the Beirut port (33.901° N, 35.519° E) shortly after 6 o’clock in the local evening (15:08:18 UT) and caused ~ 200 deaths, 6500 injuries, > 300,000 temporarily homeless people, and collective property damage of 10–15 billion US dollars^1^. This catastrophic explosion has formed ~ 140 m diameter crater at the center of the explosion (Fig. 1a). The infrasound excited by this explosion was recorded in Tunisia, Germany, and Ivory Coast, and seismic stations within ~ 500 km captured seismic waves^2^. In fact, the United States Geological Survey (USGS) has registered this explosion as a seismic event of M3.3^3^. This is considered one of the most powerful non-nuclear anthropogenic explosions in the human history^1^.

A strong explosion may significantly disturb the Earth’s upper atmosphere (Fig. 1b). Ionospheric disturbances caused by explosive events was first detected in 1958, during the International Geophysical Year, when aboveground nuclear tests were carried out on Johnston Island in the Northern Pacific^4^. Atmospheric waves were also detected during series of aboveground nuclear test by Soviet Union during 1961^5^. Such extensive aboveground testing was banned in 1963, leading to belowground tests with relatively smaller atmospheric wave amplitudes^6^. Thus, the Beirut explosion is expected to leave significant signatures in ionosphere.

Global Navigation Satellite System (GNSS), such as Global Positioning System (GPS), enabled us to observe ionospheric total electron content (TEC) from the phase differences of microwave signals in two frequencies from satellites. With the advent of continuous networks worldwide since 1990s, acoustic waves excited by such explosions have been often observed as traveling ionosphere disturbances^7^. They include the mine blasts^8^, volcanic explosions^9,10^ and North Korean underground nuclear tests^11,12^. Here we investigate the atmospheric waves and ionospheric disturbances caused by the Beirut explosion and compare their properties with past cases including its explosion energy.

## Results

### Observed changes in ionosphere

Microwaves undergo frequency-dependent delay in the ionosphere. GNSS satellites transmit microwave signals in multiple frequencies in L-band. This enables us to isolate ionospheric information by making the phase difference between the two carrier waves expressed in lengths. Such a difference is subsequently converted to TEC (1 TECU = 10^16^ electrons/m^2^), and we study time series of TEC from GNSS satellites for various ionospheric disturbances^13^.

The Beirut explosion occurred around the sunset time, when strong ionospheric irregularities known as equatorial plasma bubbles (EPBs) often develop due to the Rayleigh–Taylor plasma instability and mask subtle changes in ionosphere^14,15^. Gentile et al.^16^ showed that rates of EPBs occurrence depends on seasons and regions (Supplementary Fig. 1), i.e. the high rate concentrates in spring and autumn worldwide and also in winter in the America-Atlantic-Africa region. The EPB production rate is not high at the longitude of Beirut in early August. We also note that geomagnetic activity was low around the time of the Beirut explosion as suggested by various indices (e.g. Kp = 1.7) (Supplementary Fig. 3). We did not find EPB signatures in the VTEC around the explosion time (Supplementary Fig. 3), although moderate ionospheric scintillation signals are seen a few hours after the explosion (Supplementary Fig. 4).

**Figure 2:**
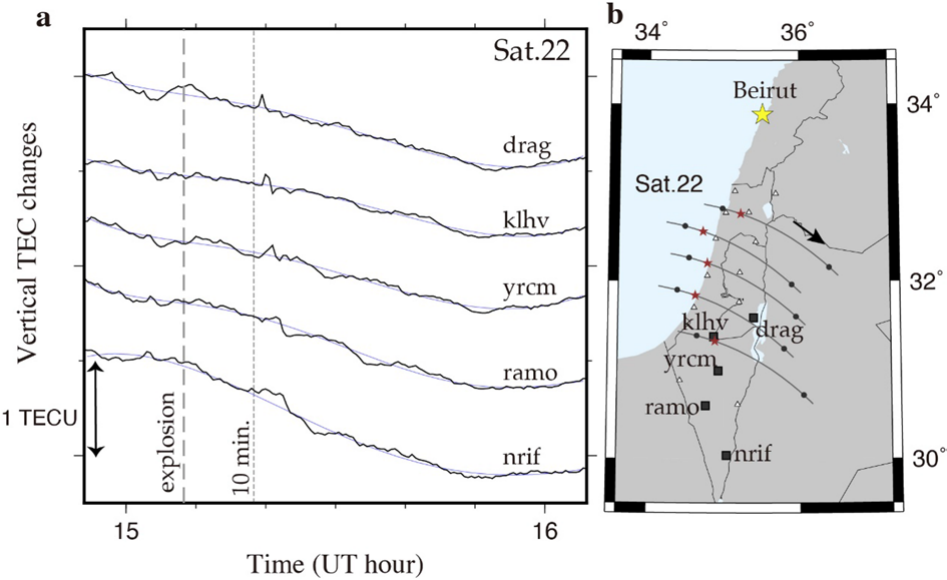
Time series of vertical TEC changes. (**a**) The VTEC change from GPS satellite 22 at stations darg, klhv, yrcm, ramo and nrif (squares in **b**) over 14:54–16:06 UT on August 04, 2020. The dashed vertical line represents the explosion time (15:08:18 UT). Ionospheric disturbances are seen shortly after the dotted line indicating 10 min after the explosion as the departure from the smoothed reference curves (best fit degree-5 polynomials, thin gray curves). (**b**) The trajectory of sub-ionospheric points (SIPs) calculated assuming the thin ionosphere at altitude 300 km, marked with solid circles (hourly time mark) and red stars (explosion time). Open triangles show other stations used to draw Fig. 3. This figure was generated using Generic Mapping Tools (version 5.2.1; URL: http://gmt.soest.hawaii.edu/).

We examined TEC data from 15 continuous GNSS stations in Israel/Palestine. Figure 2a represents vertical TEC time series obtained using the GPS satellite 22 at five ground GNSS stations to the south of Beirut. Figure 2b shows the trajectory of sub-ionospheric points (SIPs) calculated assuming the thin ionosphere at altitude 300 km. Clear N-shaped disturbances emerge 10–12 min after the explosion. The signals are invisible at stations too close to Beirut (e.g. stations to the north of drag in Fig. 2). The signals appear to arrive later with smaller amplitudes at stations farther from Beirut. We could not find clear signals for other GPS satellites (Supplementary Fig. 3). These GNSS stations also tracked GLONASS satellites, but we could not find clear signals because of unfavourable satellite distribution at the explosion time. We also note that such clear disturbance signals are either missing or ambiguous at GNSS stations far to the north, e.g. in Turkey and Cyprus. As shown in Heki^9^, such N-shaped signatures in TEC appear on the southern side of the explosion site in the northern hemisphere. Although the waves in neutral atmosphere propagates without such directivity, the electron movements are constrained along the geomagnetic field causing such directivity^17^. The disturbance amplitudes are also influenced by the difference in the angle between the wavefront and line-of-sight (LOS) of the satellites. These points are further discussed in the next section.

**Figure 3:**
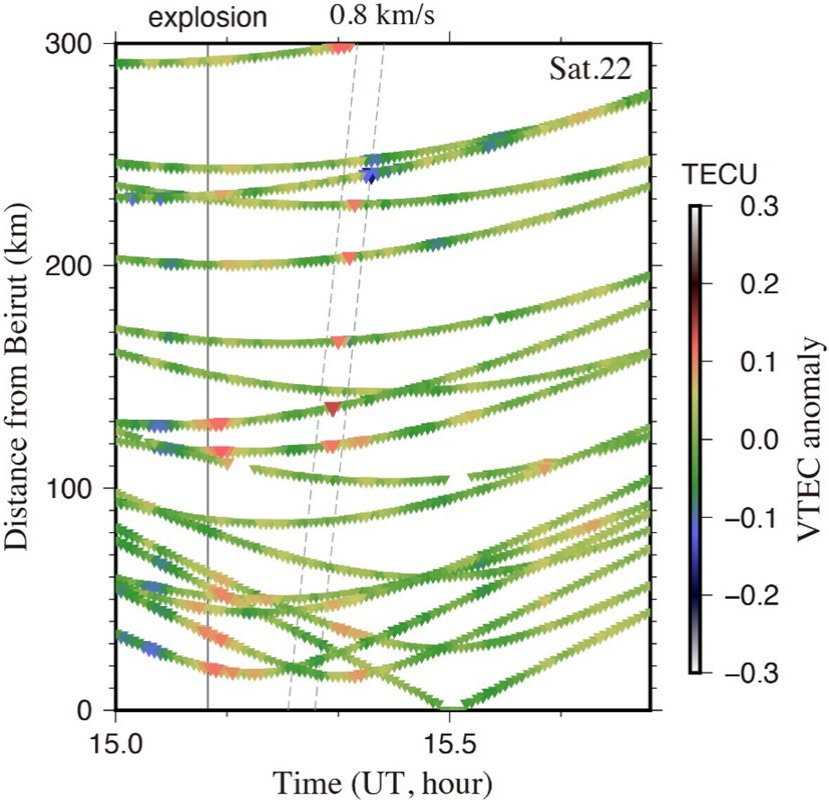
VTEC anomalies in the travel time diagram. The VTEC anomalies measured with the GPS satellite 22 are shown with colors as the function with time (horizontal axis) and distance from Beirut (vertical axis), calculated along the surface assuming the Beirut explosion point at (33.901 N, 35.519 E). The TEC disturbances by the explosion are clear for the distances exceeding ~ 120 km and propagate with the apparent velocity of 0.8 km/s (dashed lines), the sound wave speed at altitude ~ 200 km. Generic Mapping Tools (version 5.2.1; URL: http://gmt.soest.hawaii.edu/) has been used to generated this figure.

In Fig. 3, we drew a diagram indicating the distance between sub-ionospheric points (SIP) and Beirut as a function of time with colours showing the vertical TEC anomaly. There we used all the available GNSS station located to the south of Beirut. We found that the anomaly propagated southward at an apparent speed ~ 0.8 km/s. This is the sound wave velocity in the lower part of the F region of the ionosphere and much slower than the Rayleigh wave (~ 3.8 km/s) often found for coseismic ionospheric disturbances^18^, and much faster than the internal gravity wave (0.2–0.3 km/s) often excited by very large earthquakes^19^. Positive TEC anomalies seen around the explosion time within ~ 130 km from Beirut (Fig. 3) do not propagate from Beirut and would not be related to the explosion.

### Comparison with numerical simulations

**Figure 4:**
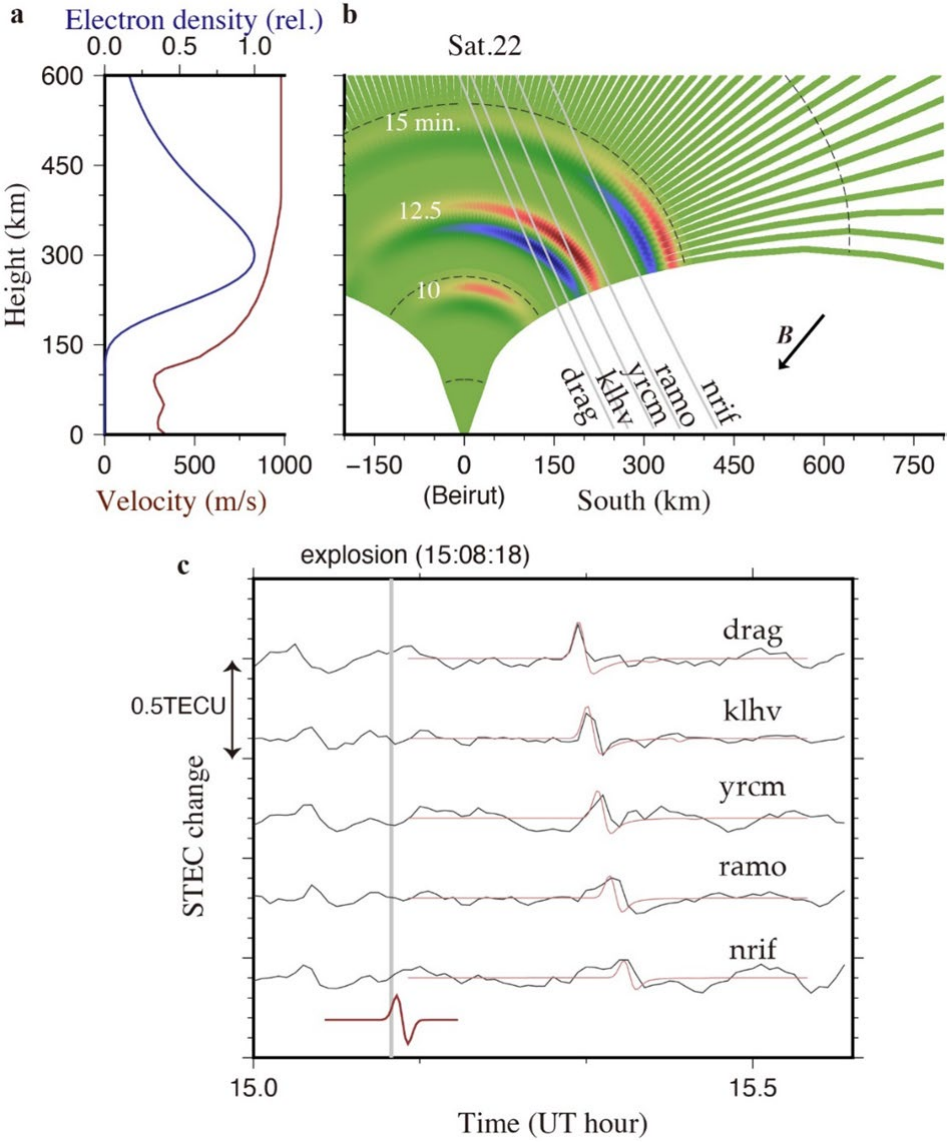
Numerical simulation of the observed TEC changes. (**a**) Relative electron density (dark blue) and velocity of acoustic wave (dark red, U.S. Standard Atmosphere 1976) plotted as functions of height. (**b**) Ray tracing of an N-shaped acoustic wave for zenith angles 0–22 degrees performed assuming the velocity profile shown in (**a**). The black dashed curves indicate equal-time contours for 5, 10, 15, and 20 min after the explosion. Colors shown along the ray paths indicate the position of the N-shaped pulse at 10.0, 12.5, and 15.0 min after the explosion. Gray lines are the LOSs 12.5 min after explosion connecting the satellite 22 and five GNSS stations (drag, klhv, yrcm, ramo, and nrif). (**c**) The observed (dark gray, residuals from the best-fit degree-7 polynomials) and synthesized (red, based on the ray tracing) STEC time series of the five GNSS stations for GPS satellite 22. Note that the observed arrival times, waveforms and relative amplitudes are consistent with the synthesized curves. Figure (**a**–**c**) was generated by using Generic Mapping Tools (version 5.2.1; URL: http://gmt.soest.hawaii.edu/).

The acoustic waves propagate upward being refracted following the velocity structure shown in Fig. 4a, and waves emitted in high angles (within ~ 20° from zenith) reach and disturb the ionospheric F-region. Here, we try to reproduce the arrival times, waveforms, and relative intensities of the observed ionospheric disturbances made by the Beirut explosion with a simple numerical simulation. Figure 4b shows the north–south vertical cross section of a space where an N-shaped acoustic pulse propagates upward. The period of the pulse was ~ 80 s (see Discussion), the period often observed in ionospheric disturbances by volcanic explosions^7,10^. Figure 4b also shows the position of the N-shaped acoustic pulse at three epochs, together with the LOSs connecting the GPS satellite 22 and five GNSS stations with clear explosion signatures in TEC. These LOSs first encounter the positive electron density anomaly 10–12 min after explosion, marking the onset of the TEC perturbation. Then LOS penetrate the negative anomaly making the TEC drops following the positive peak.

In illustrating the electron density anomalies made by the acoustic pulse, we assumed two attenuating factors, (a) height-dependence of the background electron density and (b) angle between the particle motion of neutral atmosphere and local geomagnetic field. For (a), we suppressed the electron density anomalies using a factor indicating the ratio of the electron density at that height relative to its maximum. We assumed the Chapman distribution^20^, of the altitude (*z*) dependence of the electron density,$N\left(z\right)={N}_{c}\mathrm{exp}\left(1-\varphi -{exp}^{-\varphi }\right)/2$. There, $\varphi$ = (*z = h*_c_)/*h* and the altitude of largest electron density *h*_c_ was set to 300 km, and *h* is assumed 65 km (Fig. 4a). For (b), we attenuated the anomalies by multiplying with directional cosines of the wave propagation directions onto the geomagnetic field.

Next, based on the LOS connecting the GPS satellite 22 and the five ground stations, we calculated the sum of the electron density anomaly at each intersection of the ray and LOS. By repeating the calculation every 30 s, we have synthesized slant TEC change time series and compared them with observations in Fig. 4c. The synthesized TEC are multiplied with an arbitrary factor to adjust the anomaly amplitudes (i.e. only relative amplitudes of the simulation are meaningful). Nevertheless, we can see the consistency in the ratios of TEC disturbance amplitudes among stations as well as arrival times and waveforms, between the synthesized and the observed disturbances. Although the source function has equal amounts of positive and negative anomalies, the disturbance signals are dominated by positive changes. This is because the negative electron density anomalies are partly cancelled by the penetration of the LOS through positive parts. Absence of signals at GNSS stations too close to Beirut would be due to the cancellation of positive and negative electron density anomalies caused by high-angle penetration of the LOSs with the wavefront.

## Discussions

The period of the N-shaped disturbance (~ 1.3 min) corresponds to the shortest period of the atmospheric bandpass filter (Supplementary Fig. 5), suggesting that the original energy of the atmospheric waves by the Beirut explosion concentrates in shorter-period components. The ionospheric disturbances caused by the explosion was 0.28 ± 0.03 TECU in peak-to-peak amplitude of slant TEC (calculated from the data of drag, klhv, yrcm). Supplementary Fig. 6 shows the fit of synthesized time series with the data from the two stations, drag and klhv (same as the top two in Fig. 4c). There, we changed the period of the source function and found 80 s (~ 1.3 min) best reproduces the observed waveform.

Ionospheric disturbances by five volcanic explosions of four active volcanoes, observed using GNSS-TEC data in Japan, were recently compiled^10^. The amplitudes of the slant TEC disturbance, normalized by background vertical TEC, would serve as a measure for the intensity of volcanic explosions. The background vertical TEC of the Beirut explosion is ~ 13.4 TECU according to the Global Ionospheric Map^21^ (Supplementary Fig. 7), which makes the ratio ~ 2.1%. This is comparable to recent volcanic explosions in Japan studied in Cahyadi et al.^10^, and is slightly larger than the 2004/9/1 (20:02 local time) eruption of the Asama Volcano, Central Japan, whose ionospheric disturbances were found for the first time with GNSS by Heki^9^ (Supplementary Fig. 8).

Ionospheric disturbances with peak-to-peak amplitude of ~ 0.03 TECU by a surface mine blast in Wyoming, USA in 1996 with 1.5 kt of ANFO explosives (similar in power to TNT) were detected with GPS receivers^8^. Despite a slightly larger amount of explosives, its TEC disturbance is only ~ 1/10 of the present case. Although the exact background VTEC at that time is unknown, the difference seems significant. We think this small amplitude partly comes from the larger incidence angle of the LOS connecting the ground stations in Wyoming and GPS satellite 6 with the wavefront (stations are located between the blast site and SIPs). It would also be partly due to the design of the mine blast done in a pit to fracture surface rocks, which is different from the Beirut explosion that occurred on an unguarded surface.

## Materials and methods

### GNSS and space weather data

To estimate TEC variation during August 4, 2020 Beirut explosion, we have collected RINEX GNSS data from stations in Israel and Palestine, operated and maintained by survey of Israel and archived at Scripps Orbit and Permanent Array Centre (SOPAC, http://sopac-old.ucsd.edu/dataBrowser.shtml). We use the standard sampling rate of 30 s in the daily GNSS file in order to quantify the TEC changes in the ionosphere during the Beirut explosion. We also studied data from GNSS stations in Turkey, Iraq, Cyprus, but did not find clear signals related to the explosion.

We used the Kp and Dst indices, F10.7 and changes in geomagnetic horizontal (H) and vertical (Z) components to evaluate geomagnetic activities during the studied day (Supplementary Fig. 2). The first two indices are based on geomagnetic field variations, and F10.7 indicates the changes in solar extreme ultraviolet radiation. The data are archived from OMNIWeb (https://omniweb.gsfc.nasa.gov/form/dx1.html). The geomagnetic data are from the Tihany station, Hungary (THY, 46.9° N, 17.9° E) (https://www.intermagnet.org/data-donnee/download-eng.php).

### GNSS-TEC processing strategy

In this study, we used GPS satellites to capture ionospheric disturbances. The phase difference between the two frequency, L_1_ (~ 1.5 GHz) and L_2_ (~ 1.2 GHz), of the microwave signals from satellites located ~ 20,000 km above the Earth’s surface provide information on the ionospheric electrons integrated along the LOS called slant TEC (STEC). The methods in GNSS-TEC are described in detail in e.g., Calais et al.^8^ and Heki^10^. We first remove ambiguities in carrier phase differences by letting them align with differential pseudo-ranges (codes). The observed STEC is a combination of the true TEC and satellite/receiver biases:

1 $${STEC}_{\left(observed\right)}={STEC}_{\left(true\right)}+{Bias}_{\left(satellite\right)}+{Bias}_{\left(receiver\right)}$$

We express TEC in TEC unit (TECU) (1TECU = 10^16^ electrons/m^2^), which is related to the delay as,

2 $$\Delta t=\left(\frac{40.3 \times TEC}{c}\right)\times \left[\left(\frac{{{L}_{1}}^{2}-{{L}_{2}}^{2}}{{{L}_{1}}^{2}{{L}_{2}}^{2}}\right)\right],$$

where $\Delta t$ is the difference in delay between the two frequencies.

We used satellite biases included in the header information of the Global Ionospheric Map files^21^ and determined the receiver bias using the minimum scalloping technique^22^. STEC values are often converted to absolute vertical TEC (VTEC) values by removing the inter-frequency biases in GNSS receivers and satellites and dividing by the obliquity factor $S\left(\theta \right)$^23^, that depends on the satellite elevation angle $\theta$.

3 $$VTEC={STEC}_{\left(true\right)}/S\left(\theta \right),$$

where the obliquity factor $S\left(\theta \right)$ is defined as

4 $$S\left(\theta \right)=\frac{1}{cos\beta }=\frac{1}{\sqrt{\left[1-{\left({R}_{e}cos\theta /\left\{{R}_{e}+h\right\}\right)}^{2}\right]}}.$$

The parameter *β* is the incidence angle of the LOS with the ionosphere at altitude *h*, and we used the mean radius of the Earth (6,378 km) for ${R}_{e}$.

### Modeling of GNSS-TEC time series

The arrival times, waveforms, and relative intensities of the observed ionospheric disturbances by the Beirut explosion are reproduced by adjusting a simple numerical function following Heki^9^. The function is made of a set of positive and negative pulses (red curve in Fig. 4c) and expressed as

5 $$f\left(t\right)=-\left(at\right)exp\left(\frac{-{t}^{2}}{2{\sigma }^{2}}\right),$$

where a represents amplitude of the disturbance. This numerical function has maximum and minimum at *t* =$-\sigma$ and *t* = $\sigma$ respectively, and we assumed that the explosion occurred at *t* = $-2\sigma$. We used $\sigma$ = 20, which gives 80 s (~ 1.3 min) as the period of the disturbance. As shown in Supplementary Fig. 6, this value best reproduces the observed disturbances. This period approximately corresponds to the shortest period of the band-pass filter by the atmosphere (Supplementary Fig. 5).

## Supplementary Information

Supplementary Information

## Data Availability

The GNSS and Space weather data used in this paper can be obtained online (https://doi.org/10.6084/m9.figshare.13121807). Fortran source codes used in the work are given in http://www.ep.sci.hokudai.ac.jp/~heki/software.htm.
