# Supplementary material for: Atmospheric wave energy of the 2020 August 4 explosion in Beirut, Lebanon, from ionospheric disturbances
Source: Sci Rep. 2021 Feb 2;11:2793. doi: 10.1038/s41598-021-82355-5 (PMC7854621; doi:10.1038/s41598-021-82355-5)
Supplement: Supplementary file 1 — Supplementary Information [file 41598_2021_82355_MOESM1_ESM.docx]

**Supplementary Material**

**Atmospheric wave energy of the 2020 August 4 explosion in Beirut, Lebanon, from ionospheric disturbances**

**Bhaskar Kundu^1*^, Batakrushna Senapati^1^, Ai Matsushita^2^, Kosuke Heki^2^**

^1^ Department of Earth and Atmospheric Sci., NIT Rourkela, Rourkela 769008, India.

^2^ Department of Earth and Planetary Sci., Hokkaido Univ., Sapporo, Hokkaido 060-0810, Japan

***Corresponding author:** Bhaskar Kundu, Department of Earth and Atmospheric Sciences, NIT Rourkela, Rourkela-769008, India, ([rilbhaskar@gmail.com](mailto:rilbhaskar@gmail.com))

**Supplementary Figures:**


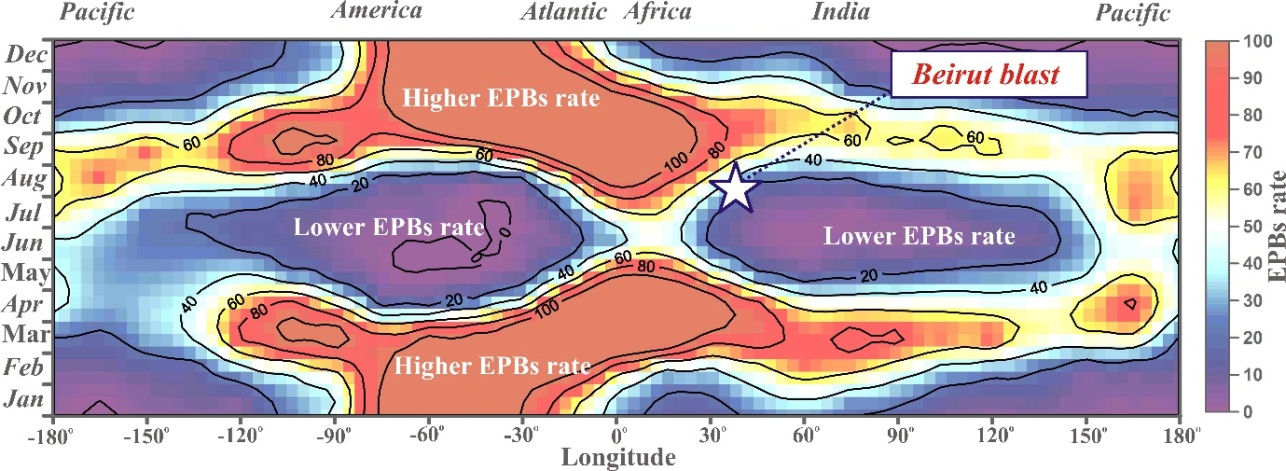


**Supplementary Figure 1.** Equatorial Plasma Bubbles rate as a function of time and longitude. Color contour plot of Equatorial Plasma Bubbles (EPBs) occurrence rate from 1989 to 2004 as a function of month and longitude**^1^**. Generation of EPB depends upon the time and the location on the globe. White star, representing the August 4 in Beirut, indicates a relatively low EPBs generation probability. This figure was generated using Surfer graphical application (version 13.6.618; URL: <https://www.goldensoftware.com/products/surfer>).

**
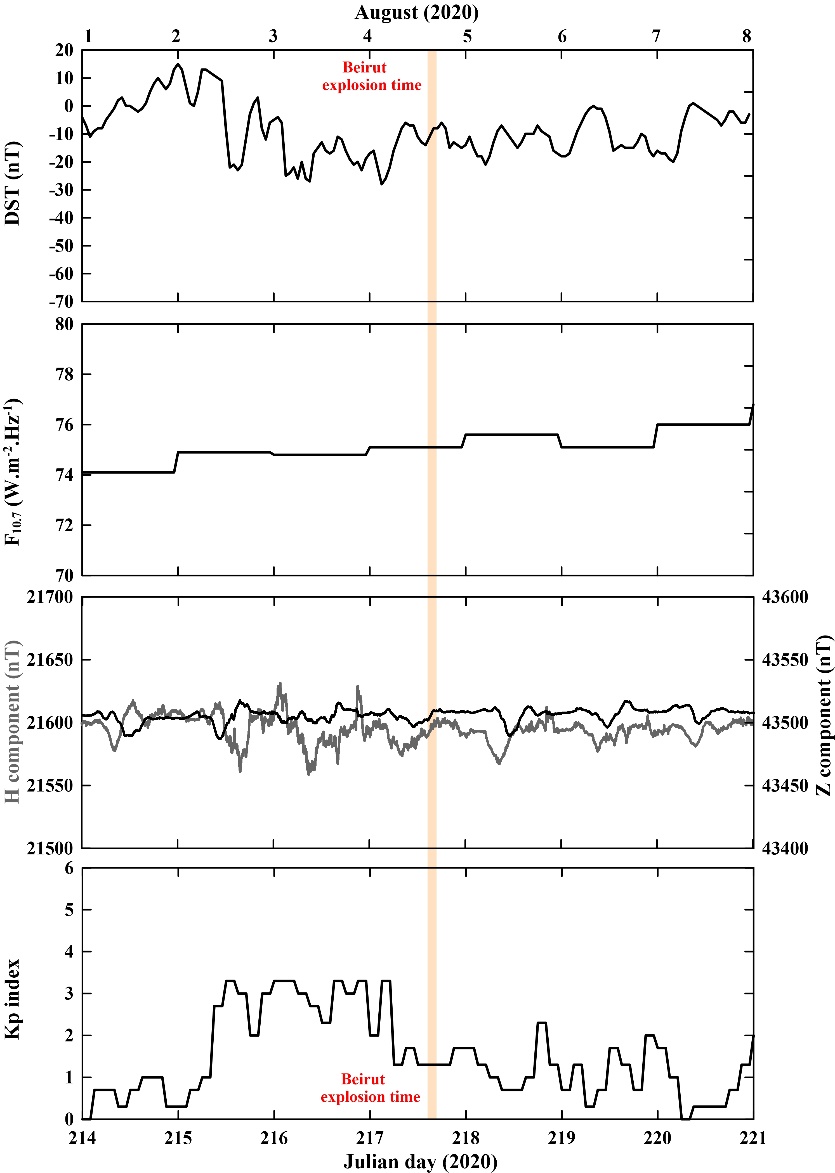
**

**Supplementary Figure 2.** Space weather around the Beirut explosion day. Here we show the geomagnetic activity indices (K_P_ Index, Dst index), F10.7 solar radiation intensity, and geomagnetic field (surface level horizontal and vertical components) from 1 to 8 August (214 to 221 Julian day) 2020, at THY in Hungary. Note the August 4, 2020 Beirut explosion occurred during a low geomagnetic activity day. This figure was generated using Grapher graphical application (version 8.7.844 URL: <https://www.goldensoftware.com/products/grapher>).


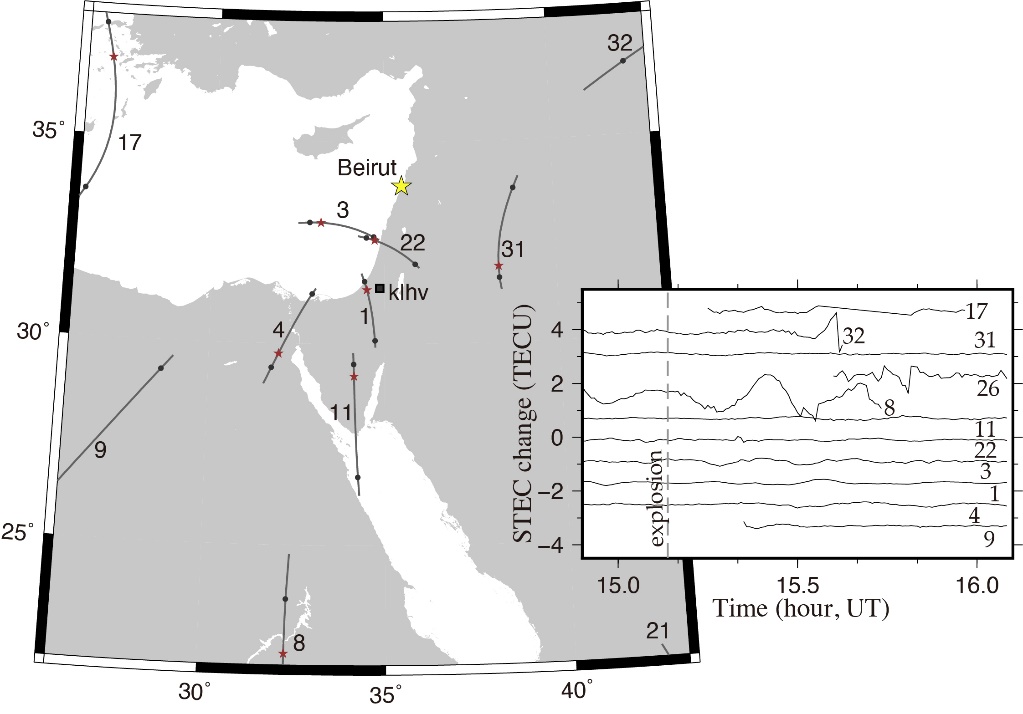


**Supplementary Figure 3.** SIPs map of GPS satellite. The black curves represent trajectories of the sub-ionospheric points (SIP) of GPS satellites from the klhv GNSS station (marked by square) during 14.9-16.1 UT. The small red stars and black circles on the SIP tracks indicate the location at the explosion time (15:08:18 UT) and hourly time marks, respectively. This station also tracked GLONASS satellites, but we could not find disturbance signals in GLONASS-TEC data. This figure was generated using Generic Mapping Tools (version 5.2.1; URL: http//gmt.soest.hawaii.edu/).


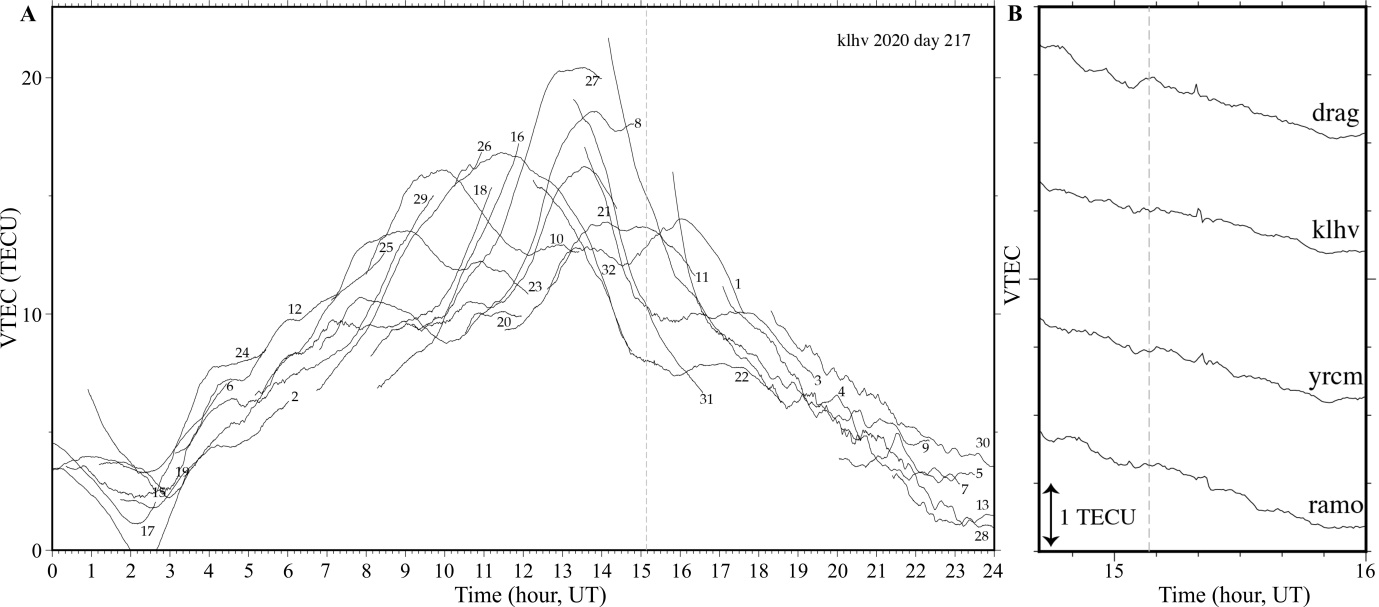


**Supplementary Figure 4.** VTEC on the explosion day at klhv. VTEC time series for all GPS satellites at the klhv GNSS station over the whole day of August 4, 2020 (day 217). Gray dashed line indicates the explosion time. We do not find any plasma bubble signatures. This figure was generated using Generic Mapping Tools (version 5.2.1; URL: http//gmt.soest.hawaii.edu/).


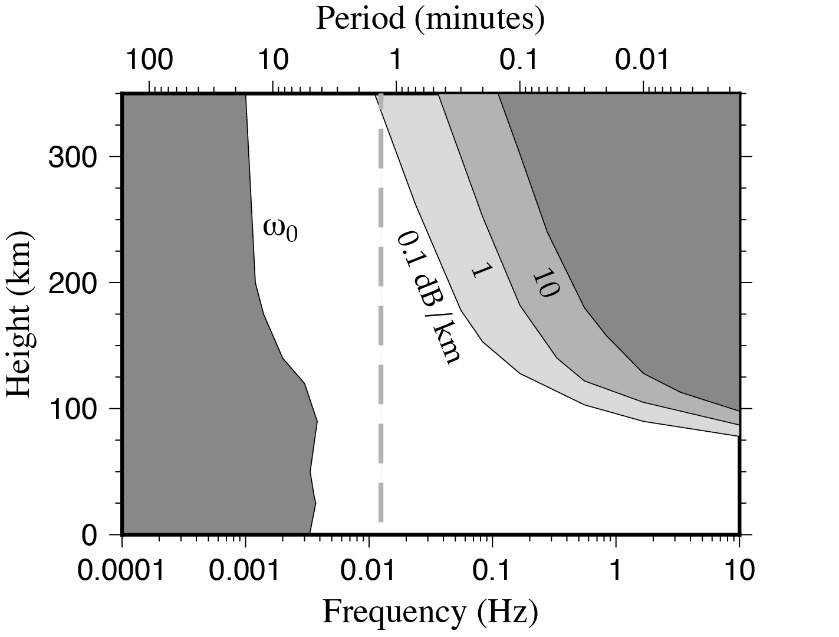


**Supplementary Figure 5.** Attenuation of airwaves in the Earth’s atmosphere. The frequency (~12.8 mHz) and period (~1.3 minutes) of the observed TEC oscillations by the Beirut explosion (vertical dashed line) and the altitude-dependent attenuation of acoustic waves in the Earth’s atmosphere, modified from Blanc**^2^**. The observed frequency corresponds to the high end of the atmospheric band pass filter**.** This figure was generated using Generic Mapping Tools (version 5.2.1; URL: http//gmt.soest.hawaii.edu/).


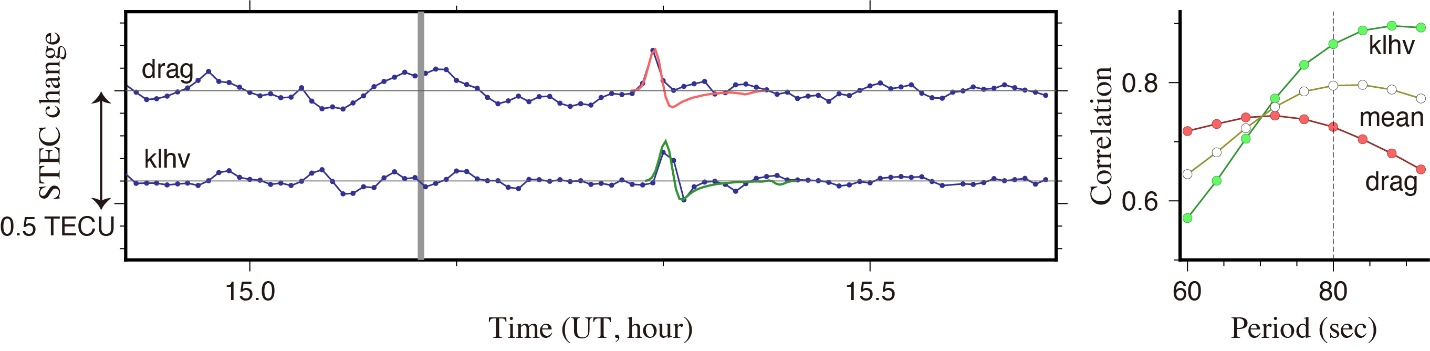


**Supplementary Figure 6.** Fit of the ionospheric disturbances caused by the source function defined with a simple form (Equation 5) to the STEC disturbances by the Beirut explosion observed at drag and klhv (departure from best-fit degree 7 polynomial). By changing the period of the source function from 60 seconds to 92 seconds (changing step is 4 seconds), we found that 80 seconds maximizes the correlation between the observed and synthesized curves of the two stations. This figure was generated using Generic Mapping Tools (version 5.2.1; URL: http//gmt.soest.hawaii.edu/).


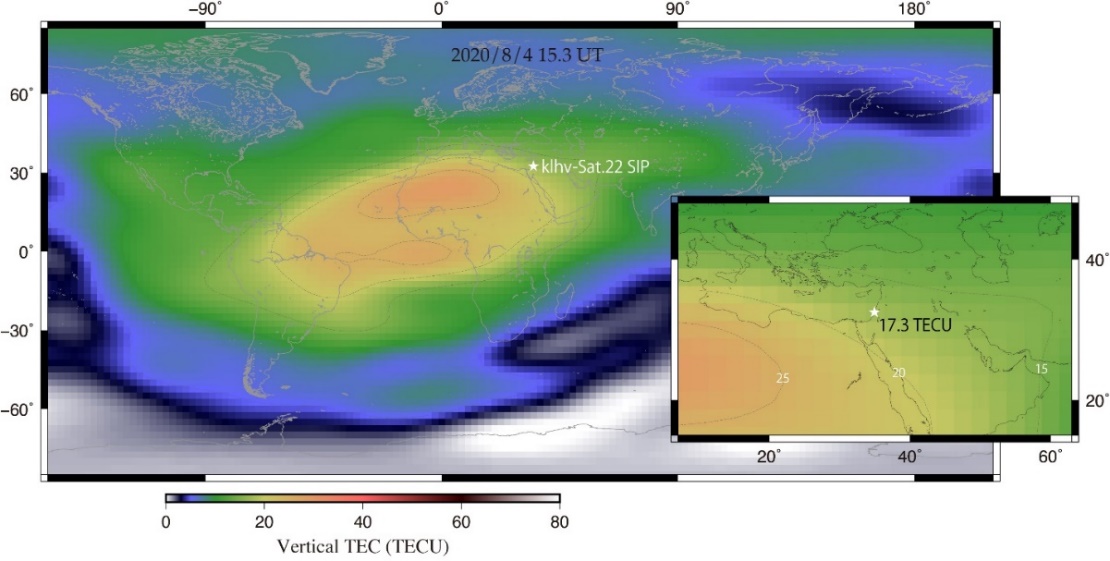


**Supplementary Figure 7.** VTEC distribution from Global Ionospheric Map**^3^**. The inset shows the close-up of the studied region. VTEC at the SIP of Satellite 22 and the station klhv at the time 15.3 UT (time of the occurrence of the disturbance) is 17.3 TECU. We applied a correction of -3.9 TECU and obtained the revised value of 13.4 TECU compensating for the poor resolution of GIM in regions of large spatiotemporal gradient in electron densities. The amount of correction is derived by comparing the values of VTEC directly from GIM and from VTEC curves obtained by using the receiver and satellite inter-frequency biases given as the GIM file header information. This correction is reflected as the error bar in Supplementary Figure 8. This figure was generated using Generic Mapping Tools (version 5.2.1; URL: http//gmt.soest.hawaii.edu/).

**
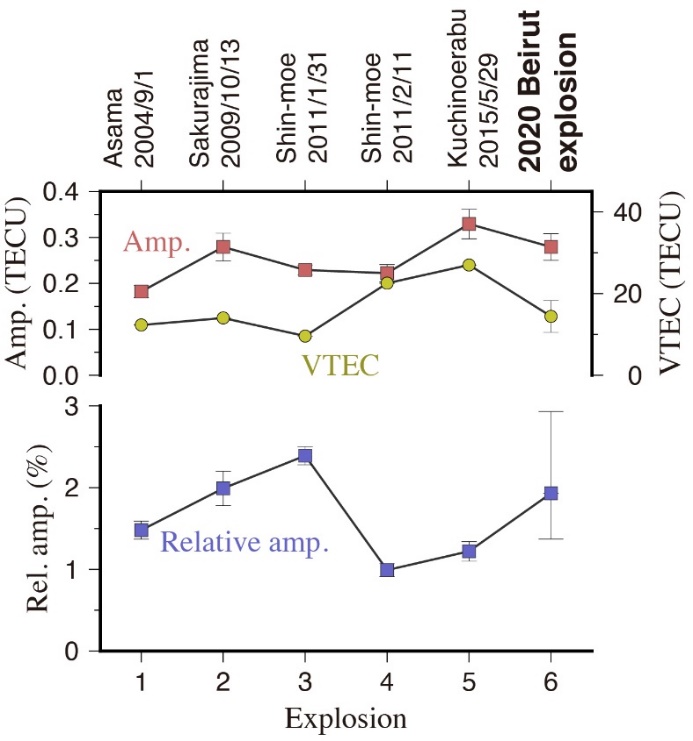
**

**Supplementary Figure 8.** Disturbance amplitude of STEC and its comparison with volcanic explosions in Japan. Comparison of the STEC amplitudes (red squares) to background VTEC (yellow circles) for the Beirut explosion and five volcanic explosions reported in Cahyadi et al.,**^4^**. Note that the airwave energy of the Beirut explosion is comparable to these recent volcanic explosions in Japan. This figure was generated using Generic Mapping Tools (version 5.2.1; URL: http//gmt.soest.hawaii.edu/).

**References:**

1. Gentile, I. C., Burke, W. C. & Rich, F. J. A global climatology for equatorial plasma bubbles in topside ionosphere. *Ann. Geophys.* ***24***, 163-172 (2006).

2. Blanc, E. Observations in the upper atmosphere of infrasonic waves from natural or artificial sources: A summary. *Ann. Geophys.* ***3*(6)**, 673–688 (1985).

3. Mannucci, A. J., Wilson, B. D., Yuan, D. N., Ho, C. H., Lindqwister, U. J. & Runge, T. F. A global mapping technique for GPS-derived ionospheric total electron content measurements. Radio Sci. 33, 565–582, (1998).

4. Cahyadi, M. N., Handoko, E. Y., Rahayu, R. W. & Heki, K. A new index for the volcanic explosion scales using impulsive ionospheric disturbances. *Adv. Space Res*. under review (2020).
